# HPV vaccine knowledge gaps and vaccination intent: a cross-sectional study of vocational students in Southern Xinjiang of China in 2023

**DOI:** 10.1186/s12889-025-25209-3

**Published:** 2025-12-03

**Authors:** Guligeina Abudurexiti, Kadeliya Muhetaer, Jing Yang, Qian Zhuo, Tingting Qi, Zikereya Saimi, Yumei Ouyang, Huilin Yu, Paizilaiti Yilamujiang, Nafeisha Adili, Nigeerayi Nuermaimaiti, Guzhalinuer Abulizi, Remila Rezhake

**Affiliations:** 1 Gynecological oncology center, Affiliated Cancer Hospital of Xinjiang Medical University, 789 Suzhou East Street, Xinshiqu District, Urumqi, 830000 China; 2 Xinjiang Key Laboratory of Oncology, Affiliated Cancer Hospital of Xinjiang Medical University, Urumqi, 830000 China; 3 School of Public Health, Xinjiang Medical University, Urumqi, 830000 China; 4 Key Laboratory of Medical Innovation Research and biomedical Transformation, Affiliated Cancer Hospital of Xinjiang Medical University, Urumqi, 830000 China

**Keywords:** Human papillomavirus (HPV), HPV vaccination, Vocational students, Awareness, Vaccination willingness, Vaccination barriers

## Abstract

**Background:**

Female students in tertiary vocational institutions, particularly in regions with limited medical resources, play a crucial role in grassroots healthcare and cervical cancer prevention. Understanding their awareness of human papillomavirus (HPV), willingness to receive the HPV vaccine, and perceived barriers is essential for promoting vaccination equity.

**Methods:**

A cross-sectional study was conducted from March to April 2023 among female vocational students in southern Xinjiang, China, using a standardized electronic questionnaire. The collected data included sociodemographic characteristics, HPV-related knowledge, vaccination willingness, and barriers. Descriptive statistics and logistic regression analyses were performed.

**Results:**

Among 1,157 participants, only 37.0% had heard of HPV, and 28.9% were aware of the HPV vaccine. Univariate analysis revealed that non-medical majors and students from rural areas had significantly lower odds of having adequate knowledge (OR = 0.450, 95% CI: 0.360–0.563, *p* < 0.001; and OR = 0.458, 95% CI: 0.350–0.600, *p* < 0.001; respectively). In contrast, higher parental education (junior college/bachelor’s degree or above) was associated with 58% greater odds of higher awareness (OR = 1.576, 95% CI: 1.119–2.219, *p* = 0.009). Additionally, students who had not heard of HPV or the HPV vaccine were significantly less likely to possess sufficient awareness (OR = 0.230, 95% CI: 0.181–0.293, *p* < 0.001; and OR = 0.194, 95% CI: 0.150–0.250, *p* < 0.001; respectively). Further, multivariate analysis confirmed that major, residence, prior HPV awareness, and vaccine awareness were independent predictors of knowledge level (all *p* < 0.001). Specifically, medical students demonstrated greater knowledge regarding HPV typing (41.8% vs. 25.6%) and optimal vaccination timing (16.7% vs. 7.9%) than non-medical students. Regarding vaccination attitudes, only 32.1% of the overall students expressed willingness to receive the HPV vaccine, with higher willingness rates observed among medical students (44.6% vs. 19.7%), urban residents (47.8% vs. 27.9%), and individuals who were aware of HPV (50.5%) or the HPV vaccine (59.3%). Actual uptake of HPV vaccine, however, remained low (1.1%–4.5%). Regression analysis showed that medical students had 2.4 times higher vaccination intent, and urban residents exhibited 1.7 times higher intent, compared to their counterparts. Furthermore, awareness was positively correlated with willingness to vaccinate, whereas an earlier age at sexual debut was linked to reduced vaccination intent (OR = 0.57, 95% CI: 0.37–0.89; *p* = 0.02). The main barrier to vaccination was the low perceived risk associated with young age (45.4%); medical students reported greater concerns about access and cost, whereas psychological barriers, such as worries about side effects and doubts about safety and efficacy, were common across all groups.

**Conclusions:**

HPV awareness and vaccination uptake among female vocational students in southern Xinjiang remain low. Targeted health education initiatives should focus on non-medical students, rural populations, and individuals from lower socioeconomic backgrounds. Enhancing vaccine accessibility and affordability through institutional and governmental efforts is critical to improving vaccination coverage in this population.

**Supplementary Information:**

The online version contains supplementary material available at 10.1186/s12889-025-25209-3.

## Background

 Cervical cancer remains a significant global health issue, ranking fourth in both incidence and mortality among malignancies affecting women. The disease burden is disproportionately higher in countries with medium or low Human Development Index (HDI) compared to those with high or very high HDI [1]. Notably, in China, the peak incidence of human papillomavirus (HPV) infection among women occurs between the ages of 20 and 24, an age range during which many women are enrolled in tertiary education [2, 3]. This highlights university students as a population at heightened risk of HPV infection and underscores the urgent need for effective prevention and treatment strategies, particularly in low- and middle-income regions.

Persistent HPV infection is the major risk factor for cervical cancer, as well as other related diseases such as genital warts [4]. As a cancer-preventing intervention, the HPV vaccine holds significant clinical value. Due to its high potential to drastically reduce incidence and mortality, HPV vaccination is considered a promising path toward the elimination of cervical cancer, making it the first cancer to be eradicated in history [5]. Currently, the National Health Commission of China recommends vaccination for females aged 9–45 years, prioritizing those aged 9–14 prior to sexual debut. Six vaccines are available, including bivalent, quadrivalent, and nine-valent options from both imported and domestic producers [6]. Despite this, national coverage remains low but is improving, from 2017 to 2022, first-dose coverage in target groups rose from 0.01% to 10.15%, third-dose from 0% to 6.21%, with eastern regions (e.g., Beijing, Shanghai) leading and western (e.g., Xinjiang) lagging [7]. By late 2023, over 30 developed regions offered free or subsidized HPV vaccines for adolescent girls, with pilot cities achieving 80% coverage by late 2022. By October 2024, free vaccination had reached approximately 40% of eligible girls nationwide [8]. However, awareness and acceptance of HPV remain significantly uneven across different regions and populations, particularly in medically underserved areas like southern Xinjiang, where vaccination rates are still low [9–11].

While many studies have examined HPV awareness and vaccine acceptance among university students and married women, research focused on students at tertiary vocational institutions is limited. Notably, tertiary vocational students are primarily aged 18–22 years, a range directly overlapping with China’s peak age of HPV infection (17–24 years) [12], highlighting their elevated risk of HPV acquisition and the urgency of targeting them for prevention. Furthermore, many of these students—especially those in medical fields such as nursing and rehabilitation in remote areas of Xinjiang, is expected to play crucial roles in frontline healthcare and grassroots public health. They are also central to advancing cervical cancer prevention efforts in southern Xinjiang. Understanding their level of awareness and willingness to receive the HPV vaccine is essential for designing effective health education campaigns and informing future vaccination strategies. Such efforts are essential to enhancing cervical cancer prevention in regions with limited medical resources and lower health literacy.

## Materials and methods

### Study design

A cross-sectional survey was conducted between March and April 2023 among vocational school students in four prefectures in southern Xinjiang. These four prefectures (Aksu, Kashgar, Hotan, and Kizilsu Kirghiz Autonomous Prefecture) were purposively selected as they are the core administrative regions of southern Xinjiang and collectively represent its predominant demographic and socioeconomic characteristics. One major vocational college was selected from each prefecture (Aksu Vocational and Technical College, Kizilsu Vocational and Technical College, Kashgar Vocational and Technical College, and Hotan Vocational and Technical College) based on the criteria of being a representative and well-established public institution with a long operational history and a comprehensive range of programs (including both medical and non-medical fields). The study aimed to evaluate awareness of HPV, knowledge of its transmission and prevention, as well as intentions regarding vaccination. The electronic questionnaire was developed using the “Questionnaire Star” platform and distributed through a multi-stage approach comprising the following steps:

1. School-level coordination: each participating school appointed two to three faculty members who received standardized training on study objectives and questionnaire administration protocols to ensure consistent implementation.
2. Stratified sampling: to ensure disciplinary balance, the study employed a stratified random sampling approach by program (medical and non-medical). The proportional allocation of questionnaires was based on the relative size (number of students) in medical and non-medical programs within each college. From each stratum, 3–5 classes were randomly selected. Within each selected class, all enrolled female students were approached and invited to participate in the study. This method ensured that every student within the chosen classes had an equal probability of being included, thereby enhancing the representativeness of the sample and eliminating selection bias at the intra-class level.
3. Informed consent procedure: all students accessed the questionnaire via a WeChat-scannable link. The first page included a detailed statement of the study’s purpose, significance, and data usage. Participants were required to check a consent checkbox before proceeding, ensuring informed consent was obtained.
4. Data collection: participants anonymously completed the self-administered survey on their mobile devices during designated class periods. Trained faculty members were present to address any questions. The digital platform automatically recorded responses while preserving participant anonymity.

### Questionnaire content

The questionnaire was independently developed by the research team, drawing upon domestic and international literature, as well as a nationwide survey on HPV vaccines among Chinese healthcare providers. Its final version was refined through consultations and discussions among professors and researchers involved in the project (Supplementary Material: Questionnaire). To enhance the accuracy of self-reported data, particularly for sensitive topics like sexual behavior and family medical history, investigators provided clear instructions to participants during the survey administration. They were specifically advised to report only clinically diagnosed conditions and confirmed sexual behaviors, thereby minimizing speculation and recall bias. The questionnaire consisted of three main sections:

1. Sociodemographic Characteristics: these include age, institutional affiliation location, academic discipline, household registration status, parental educational levels, primary household income source, monthly personal discretionary spending (amount of money the student can freely use per month, excluding tuition and basic living expenses covered by subsidies), prior exposure to HPV related information, and sexual behavior patterns.
2. HPV and Vaccination Knowledge Assessment: objective knowledge questions were scored (1 point per correct answer, 0 for incorrect), with a maximum score of 8 points. The specific questions are: target population; transmission routes; HPV-related diseases; optimal vaccination age; best timing for vaccination; HPV classification; natural clearance of HPV; prevention of HPV infection. As no participants scored above 6 points in this study, the awareness levels were categorized into low (0 points), medium (1–2 points), and high (≥ 3 points) to ensure meaningful data stratification while reflecting the actual distribution of scores observed.
3. Vaccination Behavior and Perceptions: current immunization status, intent to receive the HPV vaccine, and perceived barriers to vaccination uptake.

### Study participants

This study examines female vocational college students from four prefectures in southern Xinjiang, China. Eligible participants met the following criteria: (1) enrollment in an accredited three-year diploma program within the target region; (2) a minimum of six months of academic attendance; and (3) provision of written informed consent. Exclusion criteria included students from non-target institutions, undergraduate or graduate students, individuals who had withdrawn from their programs, and those unable to access the questionnaire via WeChat scanning.

### Data collection

Based on conservative assumptions (a proportion of 50%, a 95% confidence interval (CI), and a 5% margin of error), we calculated a minimum required sample size to be 385 female students. The 50% proportion was chosen based on the upper limit of the 95% confidence interval from a prior meta-analysis on HPV vaccine awareness among Chinese college students [9]. This conservative value was used to ensure a sufficiently large sample size for reliable estimation of key parameters, particularly regarding awareness and willingness related to the HPV vaccine among female students in tertiary vocational institutions in southern Xinjiang. We initially approached approximately 1,800 eligible female students for questionnaire distribution. A total of 1,608 questionnaires were collected (initial response rate: 89.3%). The final sample size exceeded this threshold, ensuring adequate statistical power for subsequent analyses.

### Statistical analysis

Data were analyzed using SPSS 25.0 and R software. Normally distributed continuous data were presented as mean ± standard deviation (SD) and compared using t-tests; non-normally distributed data were expressed as median (interquartile range, IQR) and analyzed with rank-sum tests; categorical variables were summarized as frequencies (percentages) and compared using chi-square or Fisher’s exact tests, with percentage distributions visualized using bar charts. For multivariate analysis, ordinal logistic regression was applied to the three-level HPV awareness outcome, including all predetermined variables. Binary logistic regression was used for vaccination intention, incorporating only variables with *P* < 0.05 from univariate analysis. Both models were adjusted for potential confounders, and results were expressed as adjusted odds ratios (ORs) with 95% confidence intervals (CIs). Forest plots were used to visualize the multivariate analysis results. A two-sided P-value < 0.05 was considered statistically significant.

## Results

### Sociodemographic characteristics of participants

In total, 1800 students were invited to complete the questionnaires, and 1608 questionnaires were collected. After excluding the incomplete and undesirable responses (175 were excluded due to response times of less than 180 s, 258 were discarded due to invalid or inconsistent responses, and 18 were removed because of unclear institutional affiliation), a data analysis was performed based on the remaining 1157 responses (effective response rate was 64.35%).

The cohort’s age distribution was balanced, with a mean age of 19.65 ± 1.27 years (range: 16–28), comprising 49.7% under 20 years and 50.3% aged 20 or older. Academic discipline was also evenly distributed, with 49.6% medical and 50.4% non-medical majors. The majority of participants (78.8%) reported rural residence, and 68.0% had an average monthly personal discretionary spending below 1,000 RMB (approximately $140). Socioeconomic profiling revealed that 65.0% of parents had attained an education level of junior high school or below, and agricultural income was the primary source of household livelihood for 63.4% of families. Regarding sexual behavior, 73.0% of respondents reported no sexual experience. Among the sexually active subgroup (*n* = 312), 173 students (approximately 55.45% of this subgroup) had their first sexual encounter before age 18, while 139 students (approximately 44.55%) reported sexual debut at age 18 or older. Current sexual activity patterns indicated that 43.27% had one sexual partner, and 8.01% reported multiple partners. Health literacy measures indicated limited HPV awareness, with only 428 (37.0%) of the 1157 participants have heard of HPV and 334 (28.9%) have heard of the HPV vaccine. Based on participants’ self - reported medical histories, 1.1% of them had confirmed HPV infection, and 1.9% reported a family history of cervical cancer or HPV infection among first-degree female relatives (Table 1).

**Table 1:** Demographic and socioeconomic characteristics of the participants (*n* = 1,157)

Characteristics		*n*	%
Age (years)	< 20	575	49.70%
	≥ 20	582	50.30%
Major	Medical	574	49.61%
	Non-medical	583	50.39%
Residence	Urban	245	21.18%
	Rural	912	78.82%
Parental educational background	Junior high school or below	752	65.00%
	High school	265	22.90%
	Junior College/Bachelor’s degree or higher	140	12.10%
Primary household income source	Government/Public institution salary	66	5.70%
	Agricultural income	733	63.35%
	Manual labor wages	103	8.90%
	Self-employed/Business income	135	11.67%
	Other sources	120	10.37%
Monthly personal expenditure (RMB)	< 1,000	787	68.02%
	1000–2000	341	29.47%
	> 2000	29	2.51%
Age at first sexual behavior (years)	< 18	173	14.95%
	≥ 18	139	12.01%
	Denial of sexual history	845	73.03%
Current number of sexual partners	≥ 2	25	2.16%
	1 partner	135	11.67%
	No current sexual activity	997	86.17%
Prior awareness of HPV	Yes	428	36.99%
	No	729	63.01%
Infected with HPV	Yes	13	1.12%
	No	1144	98.88%
Immediate female relatives with HPV infection or cervical cancer history	Yes	22	1.90%
	No	1135	98.10%
Prior awareness of the HPV vaccine	Yes	334	28.87%

### Knowledge levels

Based on the results of the univariate analysis, several factors were significantly associated with levels of HPV and vaccine awareness. Specifically, non-medical majors and students from rural areas had significantly lower odds of having adequate knowledge (OR = 0.450, 95% CI: 0.360–0.563, *p* < 0.001; and OR = 0.458, 95% CI: 0.350–0.600, *p* < 0.001; respectively). In contrast, higher parental education (junior college/bachelor’s degree or above) was associated with 58% greater odds of higher awareness (OR = 1.576, 95% CI: 1.119–2.219, *p* = 0.009). Additionally, students who had not heard of HPV or the HPV vaccine were significantly less likely to possess sufficient awareness (OR = 0.230, 95% CI: 0.181–0.293, *p* < 0.001; and OR = 0.194, 95% CI: 0.150–0.250, *p* < 0.001; respectively). Factors such as age, personal monthly expenditure, and sexual behavior-related variables did not show significant associations (Table 2).

**Table 2:** Univariate analysis of factors associated with HPV and vaccine awareness levels

Characteristics	Category	Low	Medium	High	Univariate OR (95% CI)	*p*
Age (years)	< 20	303(52.4%)	195(47.1%)	77(46.7%)		
	≥ 20	275(47.6%)	219(52.9%)	88(53.3%)	1.223 (0.981–1.523)	0.073
Major	Medical	232(40.1%)	229(55.3%)	113(68.5%)		
	Non-medical	346(59.9%)	185(44.7%)	52(31.5%)	0.450 (0.360–0.563)	< 0.001
Residence	urban	91(15.7%)	91(22.0%)	63(38.2%)		
	rural	487(84.3%)	323(78.0%)	102(61.8%)	0.458 (0.350–0.600)	< 0.001
Parental educational background	junior high school or below	395(68.3%)	264(63.8%)	93(56.4%)		
	high school	123(21.3%)	99(23.9%)	43(26.1%)	1.297 (0.994–1.692)	0.055
	junior college/Bachelor’s degree or higher	60(10.4%)	51(12.3%)	29(17.6%)	1.576 (1.119–2.219)	0.009
Primary household income source	government/public institution salary	23(4.0%)	24(5.8%)	19(11.5%)		
	agricultural income	388(67.1%)	249(60.1%)	96(58.2%)	0.422 (0.262–0.682)	< 0.001
	manual labor wages	51(8.8%)	38(9.2%)	14(8.5%)	0.475 (0.264–0.853)	0.013
	self-employed/business income	62(10.7%)	49(11.8%)	24(14.5%)	0.573 (0.328–1.001)	0.051
	other sources	54(9.3%)	54(13.0%)	12(7.3%)	0.510 (0.290–0.895)	0.019
Monthly personal expenditure (RMB)	< 1,000	406(70.2%)	274(66.2%)	107(64.8%)		
	1000–2000	155(26.8%)	136(32.9%)	50(30.3%)	1.230 (0.968–1.563)	0.091
	> 2000	17(2.9%)	4(1.0%)	8(4.8%)	1.031 (0.477–2.227)	0.938
Age at first sexual behavior (years)	< 18	94(16.3%)	57(13.8%)	22(13.3%)		
	≥ 18	61(10.6%)	54(13.0%)	24(14.5%)	1.502 (0.982–2.299)	0.061
	Denial of sexual history	423(73.2%)	303(73.2%)	119(72.1%)	1.175 (0.856–1.612)	0.318
Current number of sexual partners	≥ 2	11(1.9%)	11(2.7%)	3(1.8%)		
	1 partner	60(10.4%)	56(13.5%)	19(11.5%)	1.026 (0.467–2.255)	0.95
	No current sexual activity	507(87.7%)	347(83.8%)	143(86.7%)	0.850 (0.408–1.767)	0.663
Prior awareness of HPV	No	460(79.6%)	212(51.2%)	57(34.5%)	0.230 (0.181–0.293)	< 0.001
	Yes	118(20.4%)	202(48.8%)	108(65.5%)		
Infected with HPV	No	572(99.0%)	408(98.6%)	164(99.4%)	1.001 (0.367–2.732)	0.999
	Yes	6(1.0%)	6(1.4%)	1(0.6%)		
Immediate female relatives with HPV infection or cervical cancer history	No	570(98.6%)	405(97.8%)	160(97.0%)	0.563 (0.257–1.234)	0.152
	Yes	8(1.4%)	9(2.2%)	5(3.0%)		
Prior awareness of the HPV vaccine	No	500(86.5%)	260(62.8%)	63(38.2%)	0.194 (0.150–0.250)	< 0.001
	Yes	78(13.5%)	154(37.2%)	102(61.8%)		

*HPV and vaccination knowledge level was assessed as low (0 points), medium (1–2 points), and high (≥ 3 points) based on observed scores

**Fig. 1:**
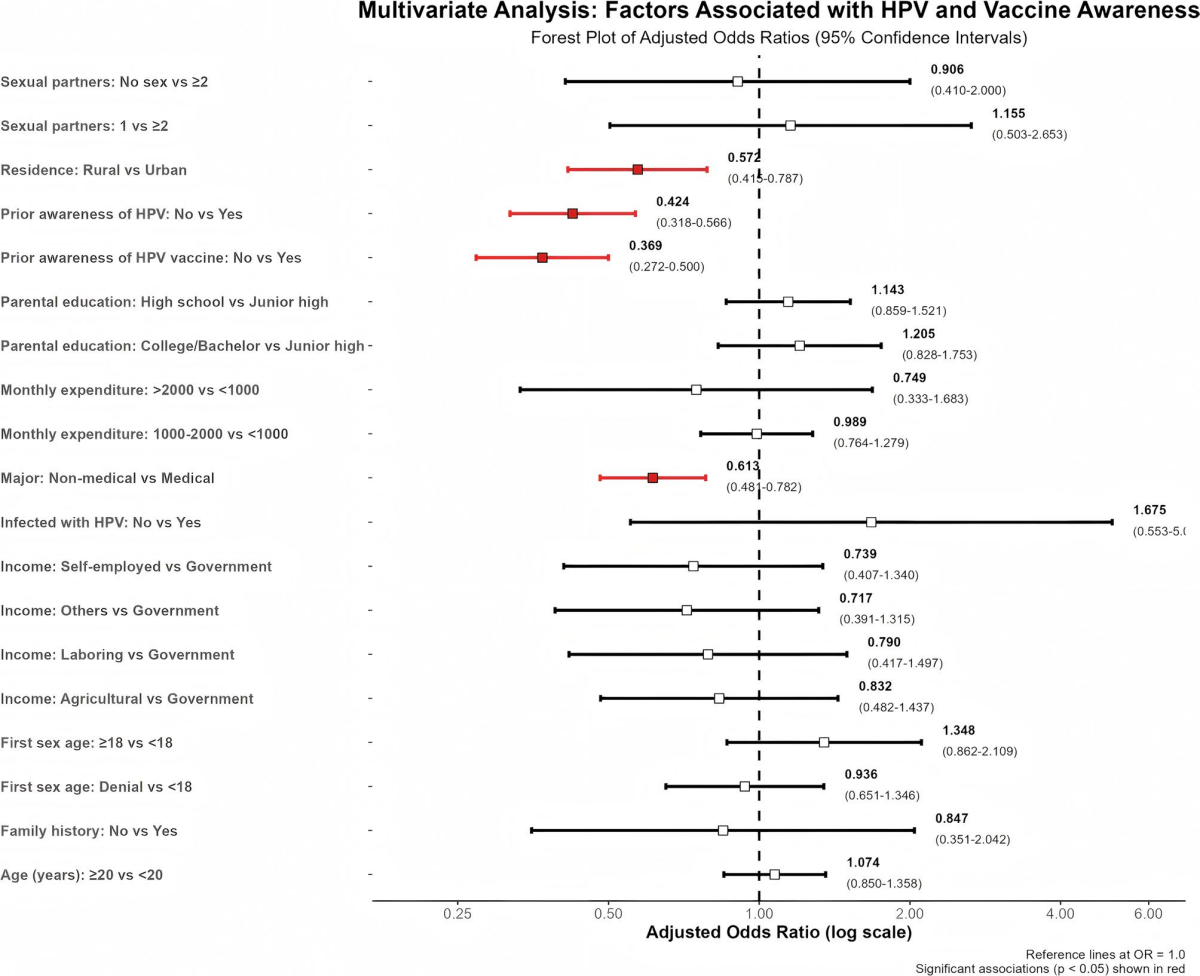
Multivariate analysis of factors associated with HPV and vaccine awareness levels

After adjusting for potential confounders in the multivariate analysis, non-medical major (aOR = 0.613, 95% CI: 0.481–0.782), rural residence (aOR = 0.572, 95% CI: 0.415–0.787), lack of prior awareness of HPV (aOR = 0.424, 95% CI: 0.318–0.566), and lack of prior awareness of the HPV vaccine (aOR = 0.369, 95% CI: 0.272–0.500) remained significant independent predictors of lower awareness (all *p* < 0.001).(Figure 1).

**Fig. 2:**
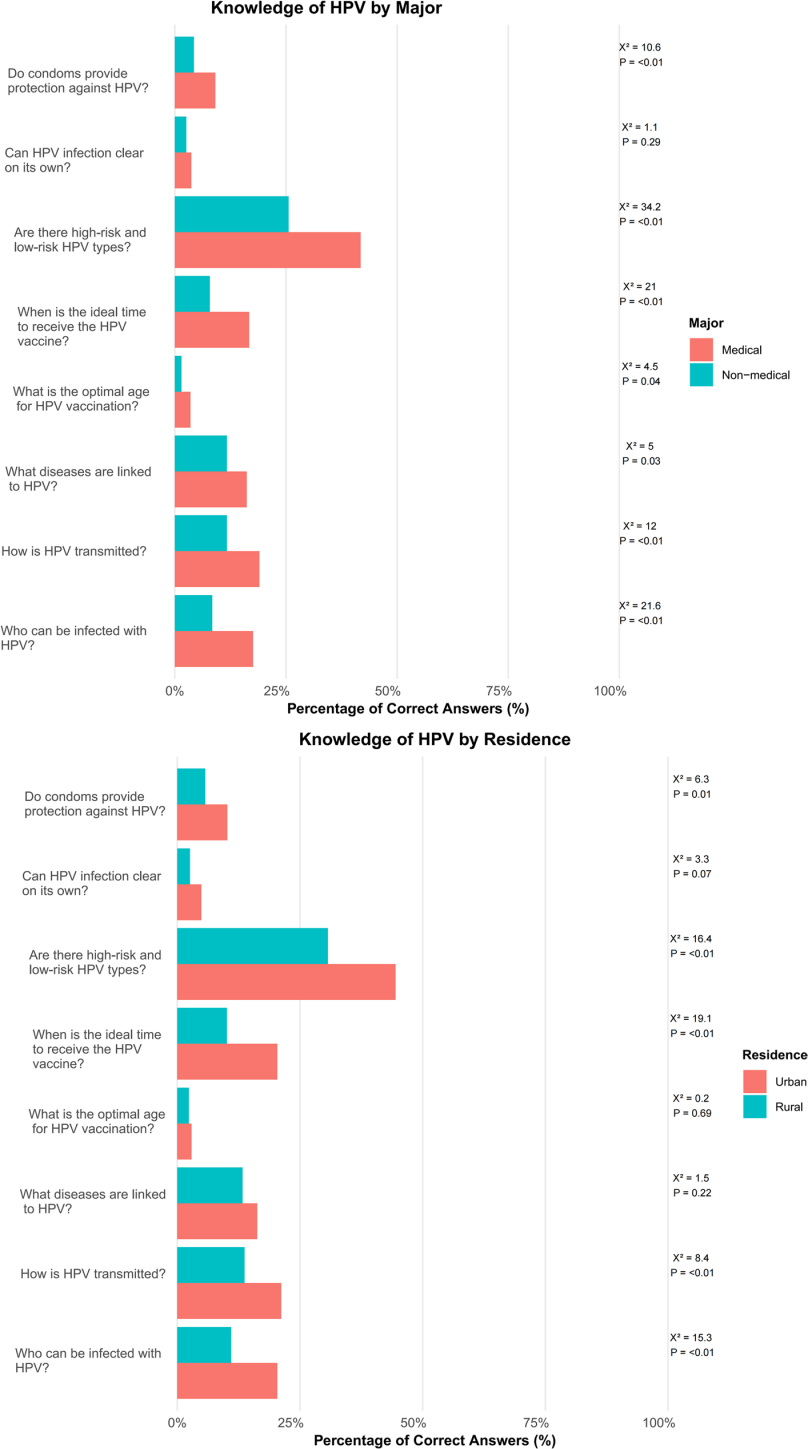
Comparison of HPV-related awareness among different groups. **A** Knowledge of HPV based on the major of the students. **B** Knowledge of HPV based on the residence of the students

Comparative analyses further revealed marked disparities in HPV knowledge between medical and non-medical students and between urban and rural populations. Medical students scored significantly higher in 6 out of 8 knowledge domains (all *p* < 0.05), particularly in understanding HPV classification (41.8% vs. 25.6%) and optimal timing of vaccination (16.7% vs. 7.9%). Similarly, urban respondents outperformed rural counterparts in 5 of 8 domains (*p* < 0.01), with the largest gaps observed in knowledge of transmission routes (21.2% vs. 13.7%) and HPV type classification (44.5% vs. 30.7%). Despite these differences, significant knowledge gaps persisted across all subgroups. Awareness of HPV’s natural clearance remained critically low (< 5%), including among medical students (3.7%) and rural residents (2.6%). Knowledge of the ideal vaccination age was especially limited among non-medical students (1.5%) and urban residents (2.9%). Furthermore, the understanding of condom efficacy in preventing HPV transmission was universally poor, with correct response rates below 10% in all groups (Fig. 2).

### Analysis of vaccination attitudes

**Fig. 3:**
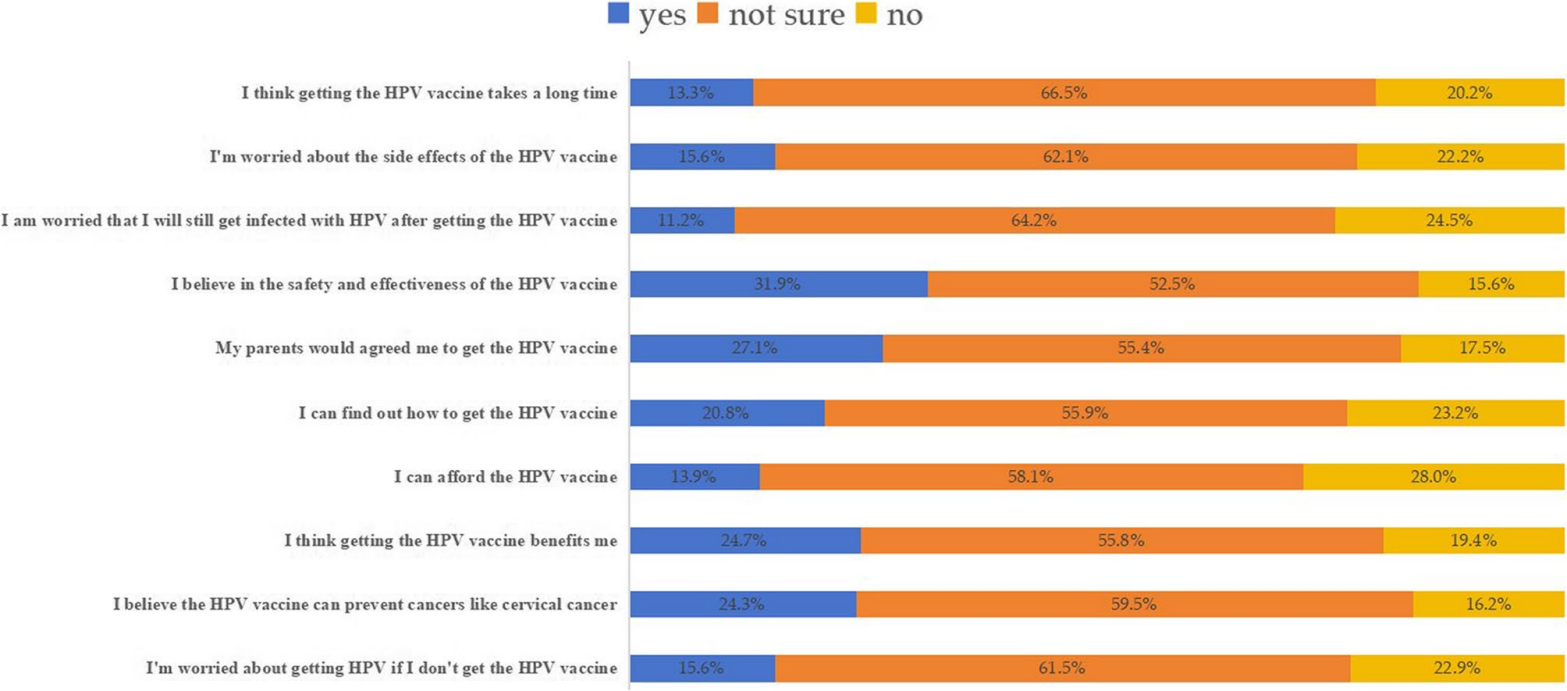
Students’ attitudes towards HPV vaccination

The survey revealed that 32.1% of students expressed willingness to receive the HPV vaccine. In terms of vaccine perception, 31.9% of respondents reported confidence in both the safety and efficacy of the vaccine, whereas a majority (55.0%) remained skeptical about its effectiveness. Notably, concerns related to potential side effects, time commitment, or financial burden were cited by fewer than 20% of participants as barriers to vaccination (Fig. 3).

Comparative analysis between medical and non-medical students revealed that medical students demonstrated significantly greater confidence in vaccine safety and effectiveness compared to their non-medical counterparts (35.4% vs. 28.5%, χ² = 6.73, *p* = 0.03) (Sup Table 1). No other statistically significant differences were observed in affirmative responses across the remaining attitude domains.

### Factors influencing HPV vaccination willingness and actual uptake rates

The study identified significant disparities in HPV vaccination willingness and actual uptake across various demographic and socioeconomic subgroups. Medical students reported a significantly higher willingness to be vaccinated (44.6%) compared to non-medical students (19.7%), while urban residents (47.8%) were more likely to express willingness than their rural counterparts (27.9%). Participants who were aware of HPV (50.5%) or the HPV vaccine (59.3%) demonstrated significantly greater intent to receive vaccination (*p* < 0.01). Despite these intentions, actual vaccination rates remained low overall, ranging from 1.1% to 4.5%. Notably, higher vaccination uptake was observed among students with a history of HPV infection or a family history of HPV-related disease (Table 3).

**Table 3:** Factors associated with HPV vaccination intention

Characteristics		Desire for Vaccination			Actual Vaccination		
		Yes	χ²	*p*	Yes	χ²	*p*
Age (years)	< 20	169 (29.4%)	3.75	0.05	6 (1.0%)	1.96	0.16
		202 (34.7%)			12 (2.1%)		
Major	Medical	256 (44.6%)	82.15	< 0.01	12 (2.1%)	2.13	0.15
	Non-medical	115 (19.7%)			6 (1.0%)		
Residence	Urban	117 (47.8%)	35.12	< 0.01	4 (1.6%)	0.01	0.91
	Rural	254 (27.9%)			14 (1.5%)		
Parental educational background	Junior high school or below	226 (30.1%)	8.88	0.01	8 (1.1%)	5.09	0.08
	High school	85 (32.1%)			5 (1.9%)		
	Junior College/Bachelor’s degree or higher	60 (42.9%)			5 (3.6%)		
Primary household income source	Government/Public institution salary	32 (48.5%)	17.25	< 0.01	1 (1.5%)	4.25	0.37
	Agricultural income	207 (28.2%)			10 (1.4%)		
	Manual labor wages	38 (36.9%)			0 (0.0%)		
	Self-employed/Business income	52 (38.5%)			4 (3.0%)		
	Other sources	42 (35.0%)			3 (2.5%)		
Monthly personal expenditure (RMB)	< 1,000	232 (29.5%)	9.22	0.01	11 (1.4%)	1.14	0.57
	1000–2000	125 (36.7%)			7 (2.1%)		
	> 2000	14 (48.3%)			0 (0.0%)		
Age at first sexual behavior (years)	< 18	33 (19.1%)	16.63	< 0.01	4 (2.3%)	5.76	0.06
	≥ 18	43 (30.9%)			5 (3.6%)		
	Denial of sexual history	295 (34.9%)			9 (1.1%)		
Current number of sexual partners	≥ 2	4 (16.0%)	3.32	0.19	0 (0.0%)	0.80	0.67
	1 partner	41 (30.4%)			3 (2.2%)		
	No current sexual activity	326 (32.7%)			15 (1.5%)		
Prior awareness of HPV	Yes	216 (50.5%)	105.59	< 0.01	10 (2.3%)	2.70	0.10
	No	155 (21.3%)			8 (1.1%)		
Infected with HPV	Yes	5 (38.5%)	0.25	0.62	2 (15.4%)	16.42	< 0.01
	No	366 (32.0%)			16 (1.4%)		
Immediate female relatives with HPV infection or cervical cancer history	Yes	11 (50.0%)	3.31	0.07	2 (9.1%)	8.31	< 0.01
	No	360 (31.7%)			16 (1.4%)		
Prior awareness of the HPV vaccine	Yes	198 (59.3%)	159.66	< 0.01	15 (4.5%)	26.42	< 0.01
	No	173 (21.0%)			3 (0.4%)		

### Multivariable logistic regression analysis of HPV vaccination intention

**Fig. 4:**
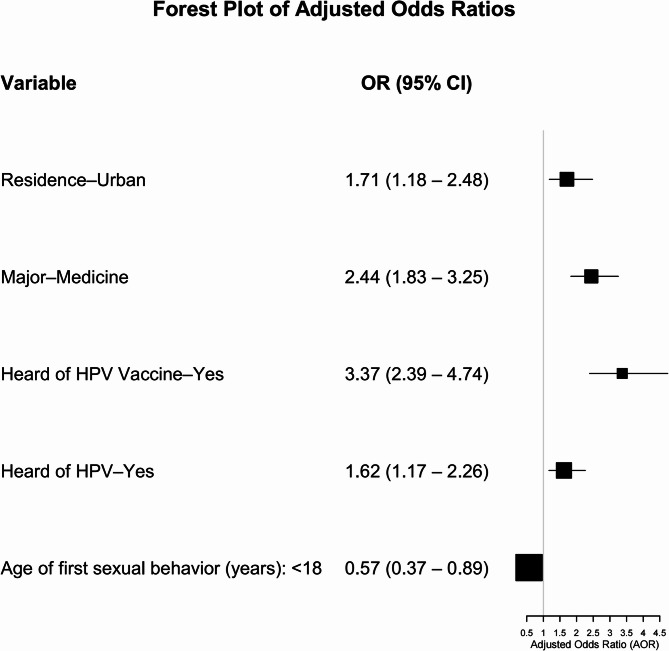
Logistic regression analysis of factors associated with HPV vaccine willingness. The forest plot displays odds ratios (ORs) and 95% confidence intervals for variables influencing students’ intent to receive HPV vaccination. The dashed vertical line represents an OR of 1. Values to the left of the line (OR < 1) indicate a negative association, while those to the right (OR > 1) indicate a positive association

The analysis revealed significant disparities in vaccination intention across subgroups. Medical students demonstrated 2.4 times higher odds of intending to receive the HPV vaccine compared to non-medical students, while urban residents exhibited 1.7 times greater intention than their rural counterparts. Awareness of HPV and its vaccine was positively associated with vaccination willingness. In contrast, early sexual debut (before age 18) was negatively associated with vaccination intent (OR = 0.57, 95% CI: 0.37–0.89; *p* = 0.02) (Fig. 4).

### Vaccination barriers

**Fig. 5:**
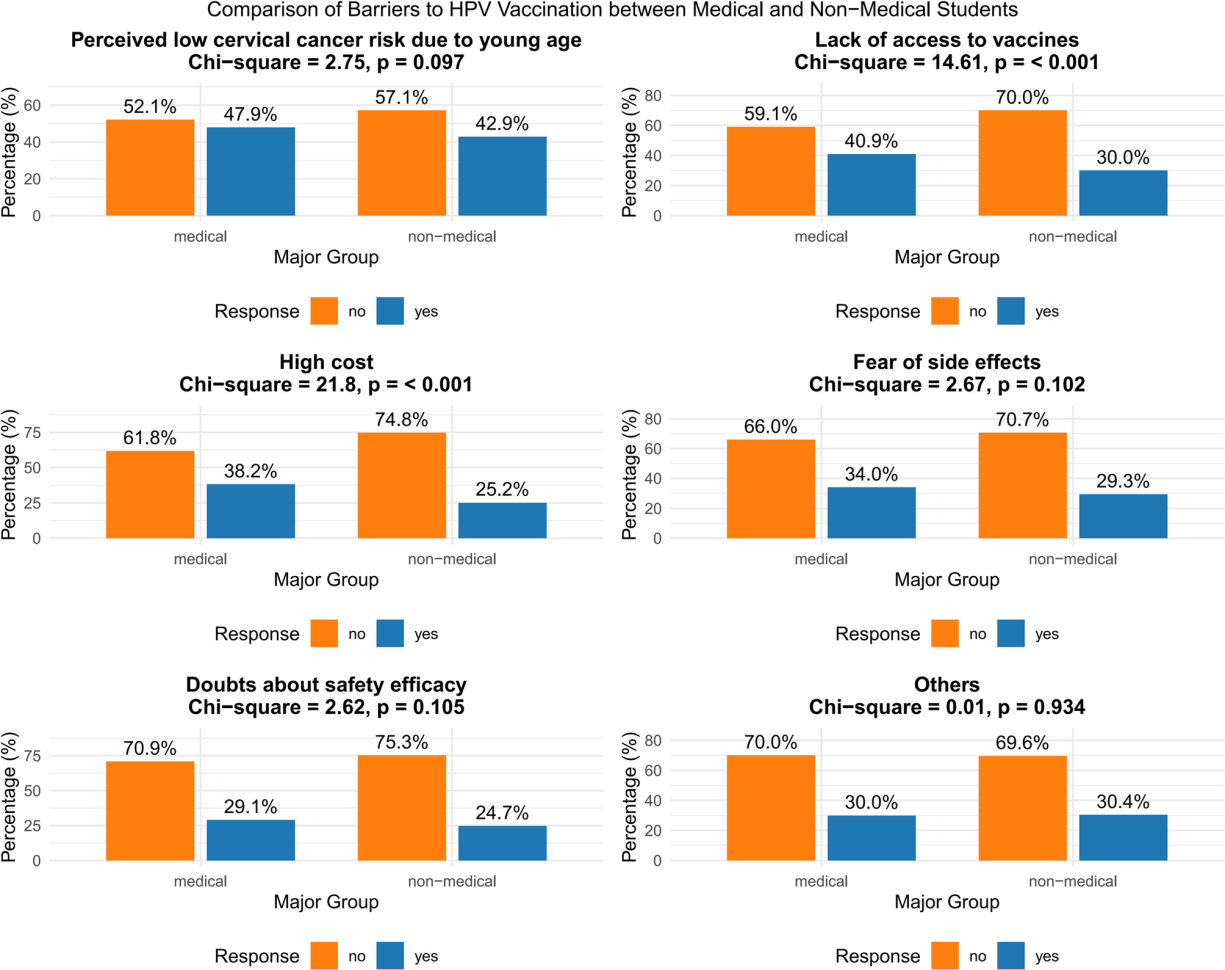
Comparison of barriers to HPV vaccination between medical and non-medical students (Chi-Square Test)

As illustrated in Fig. 5, the survey identified several key barriers to HPV vaccine uptake among students. The most frequently reported obstacle was a low perceived risk due to young age, with 45.4% of respondents believing they were “too young to develop cervical cancer.” Notably, comparative analysis between medical and non-medical students revealed significant differences in perceived barriers. Medical students reported substantially greater concerns regarding limited access to vaccination services (χ² = 14.61, *p* < 0.001) and high vaccine cost (χ² = 21.8, *p* < 0.001) compared to their non-medical counterparts. Meanwhile, psychological barriers including concerns about potential side effects (31.6%) and doubts about the vaccine’s safety and efficacy (26.9%) were consistently reported across both groups without significant differences.

## Discussion

The burden of cervical cancer remains a significant indicator of global health inequality, with approximately 84%−90% of cases occurring in low- and middle-income countries (LMICs) [13]. The World Health Organization’s (WHO) “Global Strategy to Accelerate the Elimination of Cervical Cancer” emphasizes the importance of HPV vaccination, screening, and treatment as critical components to achieve the “90-70-90” targets by 2030 [14]. Despite ongoing efforts in many LMICs, including China, vaccination coverage rates remain suboptimal [15].

Although numerous studies have examined HPV knowledge and vaccination among university students both domestically and internationally, this research differs in two key aspects: (1) Study population: students at tertiary vocational institutions differ from university students in educational background and career aspirations, necessitating a tailored approach. (2) Study location: unlike China’s top universities, concentrated in developed urban centers, vocational colleges in southern Xinjiang are situated in a geographically vast and resource-limited region, where healthcare access and health education may be insufficient. These factors likely influence students’ awareness of HPV and vaccine uptake. Understanding vaccine knowledge and coverage among these students is crucial, as they often enter grassroots healthcare roles after graduation. To our knowledge, this is the first study focusing on HPV knowledge disparities between medical and non-medical vocational students in western China, filling a critical gap in existing literature that predominantly targets university students in urban settings. By improving HPV vaccination rates in southern Xinjiang, this study aims to increase vaccine coverage, promote public health, and ultimately reduce cervical cancer incidence and mortality in underserved areas through better vaccine accessibility and health education.

This comprehensive investigation of 1,157 vocational students in southern Xinjiang unveils critical insights into disparities in HPV knowledge and barriers to vaccination within a region characterized by insufficient medical resources. While HPV awareness (37.0%) and vaccine knowledge (28.9%) among participants exceeded levels observed among vocational students in Nigeria [16], they remained markedly lower than those observed among urban university freshmen in China (59.8%) [17] and in high-income nations [18]. These findings underscore the persistent global and intra-national divide in cervical cancer prevention literacy.

These findings also reinforce prior evidence of persistent knowledge disparities, with medical students and urban residents consistently demonstrating greater awareness of HPV compared to their non-medical and rural counterparts [19]. Three major knowledge gaps emerged across the cohort: (i) limited understanding of optimal HPV vaccination timing (age and recommended window), (ii) poor awareness of HPV’s natural clearance mechanisms, and (iii) underrecognition of condom use as a preventive strategy. These gaps highlight the urgent need for coordinated interventions that combine public media campaigns with school-based health education initiatives. Vocational institutions, particularly those with medical programs, should systematically integrate comprehensive sexual health education into obstetrics and gynecology curricula, with an emphasis on practical, evidence-based topics such as condom efficacy and vaccine timing. Interestingly, while both medical and non-medical students demonstrated comparable understanding of HPV’s spontaneous clearance (noting that over 90% of infections resolve within 24 months [20]), substantial disparities remained in other domains, underscoring the informational advantage held by urban and medical-track students. Given that approximately 80% of sexually active women will contract HPV at some point in their lives [21], targeted education on the virus’s natural clearance serves a dual purpose: (1) reducing anxiety among unmarried students by promoting biomedical literacy, and (2) minimizing unnecessary medical interventions and associated costs, an especially critical goal in resource-limited settings.

This study revealed a striking discrepancy between HPV vaccination intentions (32.1%) and actual uptake (1.6%) among vocational students. Notably, all vaccinated individuals were aged 18 years or older, with medical students accounting for two-thirds of this subgroup. The observed vaccination rate lags behind that reported in mainland Chinese universities (3.1%) [15, 22] and is markedly lower than rates in high-income countries. For instance, in the U.S., HPV coverage among women aged 19–26 years reached 26.8% as early as 2011 [23], exceeding 50% by 2018 [24]. This disparity is primarily attributable to the delayed rollout and limited accessibility of the vaccine in underserved regions. Key findings indicate that vaccination willingness was significantly higher among medical students, urban residents, and individuals with prior knowledge of HPV and its vaccine. In contrast, participants who reported initiating sexual activity before the age of 18 showed lower vaccination intent. These results underscore the need for targeted interventions, both to overcome barriers among low-intent groups and to improve vaccine access among those already motivated, to effectively narrow the gap between intention and behavior.

Risk factor analysis further highlights the urgent need for targeted interventions. Students with early sexual debut, multiple partners, or a family history of HPV infection represent high-risk groups [25], yet only a small fraction within these categories reported having received the vaccine. Notably, higher vaccination uptake was observed among students with a history of HPV infection or a family history of HPV-related disease. This higher uptake may stem from infection/family history prompting greater health awareness and exposure to vaccine information via healthcare visits, though small sample sizes (only 2 vaccinated among those with prior infection) limit clarity on vaccination timing relative to infection. Previous research suggests that a family history of sexually transmitted infections (STIs) is significantly associated with HPV vaccine awareness [26]. Although no direct association between sexual activity and vaccine knowledge was observed in this study, these findings revealed the importance of focused health education. Educational initiatives should emphasize both the role of condom use in STD prevention and the protective efficacy of HPV vaccination, particularly for individuals with familial exposure to HPV-related diseases.

Moreover, our results identify multiple barriers hindering HPV vaccine uptake among students, which can be systematically categorized and addressed through targeted interventions. The most frequently cited barrier was the perception of being “too young” to require vaccination, reported by 45.4% of respondents. This misconception highlights a critical gap in health education, particularly regarding the recommended age for vaccination and the often-latent progression of HPV-related diseases. Addressing this issue requires strengthening school-based health programs through the integration of comprehensive sexual education, with an emphasis on correcting misunderstandings about HPV, promoting vaccine literacy, and encouraging timely immunization during adolescence when the vaccine is most effective. Interestingly, medical students reported significantly greater perceived barriers regarding vaccine access and cost compared to their non-medical counterparts. This may reflect their greater awareness of healthcare system limitations and economic constraints through their professional education.

Accessibility remains a major logistical challenge, particularly in geographically expansive and resource-limited regions such as Xinjiang. The sparse population distribution and limited healthcare infrastructure significantly hinder timely vaccine delivery and uptake. Nonetheless, ongoing cervical cancer elimination initiatives led by our team, along with national subsidy policies, present promising opportunities to improve vaccine accessibility in these underserved areas. A further complicating factor is the high demand for the nine-valent HPV vaccine, which has led to frequent shortages, as many individuals prefer it over more readily available alternatives. To address this imbalance, greater emphasis should be placed on promoting domestic bivalent vaccines. Notably, a 2024 study published in *Vaccines* [27] demonstrated that these bivalent formulations elicit higher antibody levels against HPV-16 and HPV-18 and offer superior cross-protection against other high-risk strains compared to quadrivalent and nine-valent options. Expanding awareness and acceptance of these vaccines could help alleviate supply constraints and ensure more equitable access to protection against HPV.

Cost remains a significant barrier to HPV vaccine uptake, with 31.6% of students citing high prices as a deterrent. In China, the three-dose nine-valent vaccine costs around 4,000 RMB (approximately $560), while the bivalent vaccine totals approximately 1,000 RMB. For students with monthly discretionary spending below 1,000 RMB, especially those in southern Xinjiang supported by national aid policies that cover basic living costs, the domestic bivalent vaccine represents the most cost-effective option. Promoting the concept that “the best available vaccine is the one you can get earliest” may shift perceptions, encourage timely vaccination, and reduce the disproportionate reliance on higher-cost variants. Affordable and accessible domestic bivalent vaccines offer a practical and budget-friendly solution to improve vaccination coverage among financially constrained student populations.

Safety concerns emerged as a significant factor influencing vaccine acceptance, with 31.6% of participants expressing worries about side effects and 26.9% expressing doubts about vaccine efficacy. These fears mirror global trends, where vaccine hesitancy in regions such as the EU and EEA is similarly driven by uncertainties regarding safety and effectiveness [28, 29]. Notably, the previously referenced 2024 study published in *Vaccines* [27] validated the excellent safety and efficacy of domestic bivalent vaccines, showing comparable performance to imported counterparts [27]. Government-led initiatives should prioritize combating misinformation, disseminating expert guidance on vaccine safety, and strengthening regulatory oversight to ensure public trust in vaccine quality. Effectively addressing these multifaceted barriers—ranging from perceived risk and access limitations to cost and safety concerns—through coordinated and targeted efforts is essential to improving HPV vaccination coverage and advancing cervical cancer prevention goals in underserved regions.

## Conclusion

In conclusion, this study underscores the urgent need for targeted interventions to improve HPV awareness and vaccination uptake among female students in tertiary vocational institutions in southern Xinjiang. Strengthening health education is critical, particularly for non-medical students, rural populations, and those with lower monthly expenditures. Educational initiatives should emphasize the complementary roles of HPV vaccination and condom use in preventing infection. To increase vaccination coverage, a multipronged approach is required, incorporating public awareness campaigns, improved vaccine accessibility, and financial support mechanisms. These strategies are especially vital in resource-limited settings like southern Xinjiang, where the burden of cervical cancer remains disproportionately high. Addressing these gaps is essential to advancing equitable cancer prevention and achieving national and global public health goals.

### Limitations and future directions

This study has several limitations. First, the use of self-designed questionnaires without formal reliability and validity assessments may limit the generalizability of the findings. Second, the inclusion of sensitive topics related to HPV infection and sexual behavior may have introduced recall bias and social expectation biases. Third, the voluntary nature of participation may have led to selection bias, with students more informed about HPV or more likely to be vaccinated being overrepresented. Fourth, the criteria for categorizing knowledge levels were based on sample-specific score distributions rather than standardized benchmarks, which may limit direct comparability with other studies. Fifth, the year of studentship was not included as a variable in this study, which limited our ability to examine the potential relationship between academic progression and HPV knowledge levels. Additionally, the cross-sectional design prevents causal inference. Future research should address these limitations by employing validated survey instruments, expanding the sample to include more diverse demographic groups, and adopting longitudinal designs to track how vocational education impacts HPV vaccine uptake over time.

## Supplementary Information

Supplementary Material 1.

Supplementary Material 2.

## Data Availability

The datasets used and analyzed during the current study are available from the corresponding author on reasonable request.

## Abbreviations

HPV: Human Papillomavirus
HDI: Human Development Index
LMICs: Low- and Middle-Income Countries
WHO: World Health Organization
STIs: Sexually Transmitted Infections
STD: Sexually Transmitted Disease
RMB: Renminbi (Chinese Yuan)
SD: Standard Deviation
OR: Odds Ratio
CI: Confidence Interval
