# Supplementary material for: HPV vaccine knowledge gaps and vaccination intent: a cross-sectional study of vocational students in Southern Xinjiang of China in 2023
Source: BMC Public Health. 2025 Dec 3;26:102. doi: 10.1186/s12889-025-25209-3 (PMC12781641; doi:10.1186/s12889-025-25209-3)
Supplement: Supplementary file 1 — Supplementary Material 1. [file 12889_2025_25209_MOESM1_ESM.docx]

**Survey on HPV Knowledge and Vaccination Intention Among Female College Students in Southern Xinjiang**

Dear Participant,

Thank you for participating in this survey. Human papillomavirus (HPV) is a virus that can cause warts and cancers. This questionnaire aims to assess students' knowledge of HPV and HPV vaccines, vaccination willingness, and influencing factors. The results will provide evidence for future HPV vaccination promotion and HPV infection prevention among students.

Confidentiality Statement:

This survey is completely anonymous.

Please answer all questions honestly based on your actual situation.

All data will be used solely for research purposes and kept strictly confidential.

By proceeding with this survey, you acknowledge that you have read this information and voluntarily agree to participate.

□ I have read and understood the above information, and I voluntarily agree to participate.

**Section 1: Demographic Information**

Age: [Single choice] *

○ 15

○ 16

○ 17

○ 18

○ 19

○ 20

○ 21

○ 22

○ 23

○ 24

○ 25

○ 26

○ 27

○ Other (please specify): _____

Current university location: [Single choice] *

○ Hotan Prefecture

○ Kizilsu Kyrgyz Autonomous Prefecture

○ Kashgar Prefecture

○ Aksu Prefecture

○ Other: _____

Major: [Single choice] *

○ Medical-related

○ Non-medical

Childhood residence: [Single choice] *

○ Urban

○ Rural

Highest parental education level (either parent): [Single choice] *

○ Junior high school or below

○ High school

○ Junior College/Bachelor’s degree or higher

Primary parental income source: [Single choice] *

○ Government/Public institution salary

○ Agricultural income

○Manual labor wages

○ Self-employed/Business income

○ Other sources: _____

Monthly personal expenditure: [Single choice] *

○ <¥1,000

○ ¥1,000–2,000

○ >¥2,000

Age at first sexual behavior (years): (Anonymous) [Single choice] *

○ ≤15

○ 16

○ 17

○ 18

○ 19

○ 20

○ 21

○ 22

○ 23

○ 24

○ 25

○ 26

○ 27

○ No sexual experience

Current number of sexual partners (concurrently): [Single choice] *

○ ≥2

○ 1

○ No sexual activity

Have you heard of HPV? [Single choice] *

○ Yes

○ No

Have you ever been infected with HPV? (Anonymous) [Single choice] *

○ Yes

○ No

Do any female blood relatives have a history of cervical cancer/HPV infection? [Single choice] *

○ Yes

○ No

Have you heard of the HPV vaccine? [Single choice] *

○ Yes

○ No

Have you received the HPV vaccine? [Single choice] *

○ Yes — _____-valent (please specify)

○ No

**Section 2: HPV/HPV Vaccine Knowledge**

Who can carry HPV? [Single choice] *

○ Females only

○ Males only

○ Both males and females

○ Unsure

HPV transmission routes (select all that apply): [Multiple choice] *

□ Sexual contact

□ Skin/mucosal contact (e.g., touching, oral contact)

□ Indirect contact (e.g., shared towels, razors, toilets)

□ Healthcare-associated transmission

□ Mother-to-child during delivery

□ Unsure

Diseases associated with HPV (select all that apply): [Multiple choice] *

□ Cervical cancer

□ Oropharyngeal/oral cancers

□ Genital warts

□ Unsure

Optimal age for HPV vaccination: [Single choice] *

○ 9–14 years

○ 15–20 years

○ >20 years

○ Unsure

Best time to get vaccinated against HPV: [Single choice] *

○ Before sexual debut

○ After sexual debut

○ Independent of sexual activity

○ Unsure

HPV can be classified as high-risk and low-risk types.

○ Yes

○ Unsure

○ No

HPV infections may resolve spontaneously.

○ Yes

○ Unsure

○ No

Condoms can prevent HPV transmission.

○ Yes

○ Unsure

○ No

**Section 3: Vaccination Willingness and Attitudes**

I would like to receive the HPV vaccine. [Single choice] *

○ Yes

○ No

○ Unsure

HPV vaccination would benefit me. [Single choice] *

○ Yes

○ Unsure

○ No

I believe the HPV vaccine prevents cervical cancer. [Single choice] *

○ Yes

○ Unsure

○ No

I worry about contracting HPV if unvaccinated. [Single choice] *

○ Yes

○ Unsure

○ No

I can afford the HPV vaccine. [Single choice] *

○ Yes

○ Unsure

○ No

I know how to access HPV vaccination services. [Single choice] *

○ Yes

○ Unsure

○ No

My parents would support my HPV vaccination. [Single choice] *

○ Yes

○ Unsure

○ No

I trust the safety and efficacy of HPV vaccines. [Single choice] *

○ Yes

○ Unsure

○ No

I worry about HPV infection despite vaccination. [Single choice] *

○ Yes

○ Unsure

○ No

I’m concerned about vaccine side effects. [Single choice] *

○ Yes

○ Unsure

○ No

The multi-dose schedule is time-consuming. [Single choice] *

○ Yes

○ Unsure

○ No

Barriers to HPV vaccination among students (select all that apply): [Multiple choice] *

□ Perceived low cervical cancer risk due to young age

□ Lack of access to vaccines

□ High cost

□ Fear of side effects

□ Doubts about safety/efficacy

□ Other: _____

How did you learn about the HPV vaccine? [Multiple choice] *

□ Friends/family

□ Media (TV, WeChat, Weibo, Tiktok)

□ School campaigns

□ Other: _____

□ Learned through this survey

What is your acceptable price per dose of the HPV vaccine? [Single choice] *

○ <¥100

○ ¥200–500

○ ¥500–1,000

○ ¥1,000–1,500
