# Supplementary material for: HPV vaccine knowledge gaps and vaccination intent: a cross-sectional study of vocational students in Southern Xinjiang of China in 2023
Source: BMC Public Health. 2025 Dec 3;26:102. doi: 10.1186/s12889-025-25209-3 (PMC12781641; doi:10.1186/s12889-025-25209-3)
Supplement: Supplementary file 2 — Supplementary Material 2. [file 12889_2025_25209_MOESM2_ESM.docx]

# Title page

**HPV vaccine knowledge gaps and vaccination intent: A cross-sectional study of vocational students in Southern Xinjiang of China in 2023**

Guligeina Abudurexiti^1,2#^, Kadeliya Muhetaer^1,2#^, Jing Yang^1,2^, Qian Zhuo^1,2^, Tingting Qi^1,2^, Zikereya Saimi^3^, Yumei Ouyang^1^, Huilin Yu^1^, Paizilaiti Yilamujiang^1^, Nafeisha Adili^1^, Nigeerayi Nuermaimaiti^1^, and Guzhalinuer Abulizi ^1,2^*, Remila Rezhake^1,4^*

1. Gynecological oncology center, Affiliated Cancer Hospital of Xinjiang Medical University, Urumqi, 830000, China
2. Xinjiang Key Laboratory of Oncology, Affiliated Cancer Hospital of Xinjiang Medical University, Urumqi, 830000, China
3. School of Public Health, Xinjiang Medical University, Urumqi, 830000, China
4. Key Laboratory of Medical Innovation Research and biomedical Transformation, Affiliated Cancer Hospital of Xinjiang Medical University, Urumqi, 830000, China

# Co-first authors

*Co-corresponding authors

**Corresponding authors:**

Prof. Guzhalinuer Abulizi, Affiliated Cancer Hospital of Xinjiang Medical University 789 Suzhou East Street, Xinshiqu District, Urumqi, 830000, China; Phone: +86-0991-7819262; Fax: +86-0991-7819262; Email: [gzlnr@qq.com](mailto:ramila513@126.com); [00157@xjmu.edu.cn](mailto:%20Remila@xjmu.edu.cn)

Prof. Remila Rezhake, Affiliated Cancer Hospital of Xinjiang Medical University 789 Suzhou East Street, Xinshiqu District, Urumqi, 830000, China; Phone: +86-0991-7589491; Fax: +86-0991-7589491; Email: [ramila513@126.com](mailto:ramila513@126.com); [Remila@xjmu.edu.cn](mailto:%20Remila@xjmu.edu.cn)

**Sup Table 1. Attitudes Toward HPV Vaccination Among Medical and Non-Medical Students *(Results compare YES responses for each attitude question)***

| **Question-Attitude** | **Medical Group** | | | **Non-Medical Group** | | | **Results** | |
| --- | --- | --- | --- | --- | --- | --- | --- | --- |
|  | **Yes** | **Not_Sure** | **No** | **Yes** | **Not_Sure** | **No** | **χ²** | **p** |
| Worried about getting HPV without vaccine | 96 (16.7%) | 342 (59.6%) | 136 (23.7%) | 84 (14.4%) | 370 (63.5%) | 129 (22.1%) | 2.02 | 0.36 |
| Believe vaccine prevents cancer | 155 (27%) | 335 (58.4%) | 84 (14.6%) | 126 (21.6%) | 354 (60.7%) | 103 (17.7%) | 5.38 | 0.07 |
| Think vaccine benefits me | 136 (23.7%) | 332 (57.8%) | 106 (18.5%) | 150 (25.7%) | 315 (54%) | 118 (20.2%) | 1.71 | 0.43 |
| Can afford vaccine | 72 (12.5%) | 324 (56.4%) | 178 (31%) | 89 (15.3%) | 348 (59.7%) | 146 (25%) | 5.74 | 0.06 |
| Can find how to get vaccine | 108 (18.8%) | 319 (55.6%) | 147 (25.6%) | 133 (22.8%) | 329 (56.4%) | 121 (20.8%) | 5.20 | 0.07 |
| Parents would agree | 166 (28.9%) | 318 (55.4%) | 90 (15.7%) | 148 (25.4%) | 323 (55.4%) | 112 (19.2%) | 3.40 | 0.18 |
| Believe in safety/effectiveness | 203 (35.4%) | 290 (50.5%) | 81 (14.1%) | 166 (28.5%) | 318 (54.5%) | 99 (17%) | 6.73 | **0.03** |
| Worried about infection after vaccine | 57 (9.9%) | 371 (64.6%) | 146 (25.4%) | 73 (12.5%) | 373 (64%) | 137 (23.5%) | 2.19 | 0.33 |
| Worried about side effects | 81 (14.1%) | 352 (61.3%) | 141 (24.6%) | 99 (17%) | 368 (63.1%) | 116 (19.9%) | 4.52 | 0.10 |
| Think vaccination takes long time | 86 (15%) | 381 (66.4%) | 107 (18.6%) | 68 (11.7%) | 388 (66.6%) | 127 (21.8%) | 3.81 | 0.15 |
